# Interval Colorectal Cancers following Guaiac Fecal Occult Blood Testing in the Ontario ColonCancerCheck Program

**DOI:** 10.1155/2016/4768728

**Published:** 2016-05-08

**Authors:** Lawrence Paszat, Rinku Sutradhar, Jill Tinmouth, Nancy Baxter, Linda Rabeneck

**Affiliations:** ^1^Institute for Clinical Evaluative Sciences and the University of Toronto, 2075 Bayview Avenue, Toronto, ON, Canada M4N 3M5; ^2^Division of Gastroenterology, Sunnybrook Health Sciences Centre and the University of Toronto, 2075 Bayview Avenue, Toronto, ON, Canada M4N 3M5; ^3^Department of Surgery, St. Michael's Hospital and the University of Toronto, 30 Bond Street, Toronto, ON, Canada M5B 1W8; ^4^Cancer Care Ontario and the University of Toronto, 620 University Avenue, Toronto, ON, Canada M5G 2L7

## Abstract

*Background. *This work examines the occurrence of interval colorectal cancers (CRCs) in the Ontario ColonCancerCheck (CCC) program. We define interval CRC as CRC diagnosed within 2 years following normal guaiac fecal occult blood testing (gFOBT).* Methods.* Persons aged 50–74 who completed a baseline CCC gFOBT kit in 2008 and 2009, without a prior history of CRC, or recent colonoscopy, flexible sigmoidoscopy, or gFOBT, were identified. Rates of CRC following positive and normal results at baseline and subsequent gFOBT screens were computed and overall survival was compared between those following positive and normal results.* Results.* Interval CRC was diagnosed within 24 months following the baseline screen among 0.16% of normals and following the subsequent screen among 0.18% of normals. Interval cancers comprised 38.70% of CRC following the baseline screen and 50.86% following the subsequent screen. Adjusting for age and sex, the hazard ratio (HR) for death following interval cancer compared to CRC following positive result was 1.65 (1.32, 2.05) following the first screen and 1.71 (1.00, 2.91) following the second screen.* Conclusion.* Interval CRCs following gFOBT screening comprise a significant proportion of CRC diagnosed within 2 years after gFOBT testing and are associated with a higher risk of death.

## 1. Introduction

Large randomized control trials have demonstrated that periodic colorectal screening by guaiac fecal occult blood testing (gFOBT) for early detection of asymptomatic colorectal cancer (CRC) reduced mortality due to CRC among screened persons compared to controls, likely due to the higher proportion of early stage CRCs observed among the screened [1]. Not all persons with an asymptomatic CRC will test positive by gFOBT. Despite a normal gFOBT result, some screened persons will receive a diagnosis of CRC prior to the next periodic screen: such CRCs are labelled as interval CRC [2, 3].

Interval CRC may represent biological variability among CRCs, possibly associated with the propensity to bleed [3]. It is unclear from the literature whether interval cancers are associated with variability in laboratory procedures although it is theoretically possible. Interval CRCs are associated with higher all-cause mortality compared to CRC diagnosed following positive gFOBT [3, 4]. It is unclear whether mortality following interval CRC diagnosis is better than, or equivalent to, mortality following CRC diagnosis among unscreened persons, because the limited published data do not agree [3, 4]. It has been recommended that colorectal screening programs monitor interval CRC arising among screenees because of the association with higher all-cause mortality [5].

ColonCancerCheck (CCC) is the provincial colorectal screening program managed by Cancer Care Ontario. CCC has recommended biennial guaiac fecal occult blood testing (gFOBT) for persons aged 50–74 years, who do not have a first-degree relative with colorectal cancer, since April 1, 2008. Opportunistic colorectal screening, primarily by colonoscopy, has existed for many years and coexists with CCC. CCC supplies primary care physicians with program-branded gFOBT kits (Hema-Screen, Immunostics Inc.) to distribute to eligible asymptomatic persons; a small number of eligible persons request a gFOBT kit from pharmacies or from a telephone information line. Each kit consists of 3 stool collection cards; persons are instructed to collect two samples per card, from 3 consecutively passed stools [6]. Kits are processed at seven community laboratories in Ontario and in accordance with published Cancer Care Ontario gFOBT laboratory standards [7]. CCC maintains a database of gFOBT results, identified by the unique numeric Ontario Health Insurance Number (OHIN) of the person who submitted the CCC gFOBT kit, the date of laboratory analysis, and the result.

The goal of this paper is to analyze interval CRC following baseline and first subsequent gFOBT screening among participants in CCC.

## 2. Methods

This work was approved by the Research Ethics Board of Sunnybrook Health Sciences Centre and conducted at the Institute for Clinical Evaluative Sciences (ICES).

The CCC gFOBT database was received at ICES, where the OHIN was encrypted. The observations with positive or normal results in the database, with laboratory analysis dates from April 1, 2008, to December 31, 2009, were linked by the encrypted identifier to the Registered Persons Database (RPDB), a roster of all beneficiaries of the universal single payer Ontario Health Insurance Plan (OHIP), to the OHIP physician billing claims database, and to the Ontario Cancer Registry (OCR). These datasets were linked using unique encoded identifiers and analyzed at the Institute for Clinical Evaluative Sciences (ICES).

In order to exclude persons who were ineligible for screening because of recent testing or prior diagnosis of CRC, the OHIP database and the OCR were searched to identify and exclude those with a billing claim for colonoscopy ≤ 10 years prior, flexible sigmoidoscopy ≤ 5 years prior, or gFOBT ≤ 2 years prior, to the date of CCC gFOBT, and those with a prior diagnosis of CRC in the OCR (International Classification of Diseases version 9 (ICD9) 153X, 1530–1534, 1536–1539, 1540-1541).

We identified the baseline gFOBT screen among those remaining observations, with a positive or normal gFOBT result dated from April 1, 2008, to December 31, 2009. These observations were linked to the RPDB for abstraction of age and sex on the date of the gFOBT screen. Those with a diagnosis of CRC occurring within 24 months of the date of the positive or normal result were identified in the OCR database and their diagnosis date and ICD9 diagnosis codes were extracted.

We identified the first subsequent gFOBT screen among those with a baseline normal result who did not have a CRC diagnosis within 24 months and who had a subsequent gFOBT screen, associated with a positive or normal gFOBT result, 21 to 30 months following the baseline screen. We searched OCR database for CRC ICD9 diagnosis codes and diagnosis dates within 24 months following the subsequent screen.

The baseline gFOBT result could have been dated between April 1, 2008, and December 31, 2009. Following a baseline normal, the latest date for a subsequent gFOBT result could have been 2.5 years (30 months) following December 31, 2009, that is, June 30, 2012. The latest date for a colorectal cancer diagnosis within 24 months following a subsequent gFOBT result therefore could have been June 30, 2014. The gFOBT database was first accessed on April 8, 2015. The OHIP and RPDB databases were first accessed on April 15, 2015. For purposes of excluding those persons with prior diagnoses of colorectal cancer, the OCR file was accessed on April 17, 2015. An updated OCR file including diagnoses confirmed during calendar year 2014 was first accessed on July 30, 2015, in order to ascertain colorectal cancers diagnosed within 2 years following positive and negative results at the baseline and subsequent screens.

For each CRC diagnosed following the baseline and subsequent gFOBT screens, we used the fourth digit of the ICD9 diagnosis codes for CRC, to dichotomize the location of the CRC as (1) distal (descending colon 1532, sigmoid 1533, rectosigmoid 1540, and rectum 1541) versus (2) proximal (cecum 1534, ascending colon 1536, hepatic flexure 1530, transverse colon 1531, and splenic flexure 1537) or unknown (153X, 1538, and 1539). CRCs diagnosed within 24 months following a normal result at either the baseline or subsequent screen are considered to be interval CRCs. Among all those with a diagnosis of CRC, we determined vital status (alive versus dead from any cause) on the date of last contact, from the RPDB.

We tabulated the count of persons with positive and normal results at the baseline and subsequent gFOBT screens by five-year age categories, stratified by sex. We tested the difference in positive results between males and females stratified by age at each screen using chi-square tests. We tabulated the count of persons with a diagnosis of CRC within 24 months following the date of the baseline and subsequent gFOBT screens, by sex, by positive versus normal result, and by distal versus another location of the colorectal cancer. We tested the difference in CRC occurrence within 24 months following the baseline and subsequent gFOBT screens between males and females, stratified by positive or normal result, using chi-square tests. We tested the difference in CRC location (distal anatomic site versus other sites) between males and females using chi-square tests.

Time from the date of CRC diagnosis among screenees to the date of death from any cause was examined using a Cox proportional hazards regression model. The model was run separately for those diagnosed after the baseline and the subsequent screens. The main exposure in the model was the gFOBT screen result (positive versus normal); the model was adjusted for age as a continuous variable, sex, and anatomic site of CRC (distal versus other). A Cox proportional hazards regression model was also implemented on the combined cohort of cancers diagnosed after either the baseline or the subsequent screen. In this model, we tested, via an interaction term, whether the association between the screen result (positive versus normal) and the hazard of death varied between the baseline and subsequent screens. 

## 3. Results

### 3.1. Baseline gFOBT Screen

From April 1, 2008, to December 31, 2009, 307,456 persons with no history of CRC and without a colonoscopy within the prior 10 years, flexible sigmoidoscopy within 5 years, or a gFOBT test within 2 years completed a CCC gFOBT kit and received a normal or a positive result. Females comprised 169,245/307,456 (55.05%) of persons completing the baseline gFOBT screen (Table 1). Within each age group, males were more likely than females to receive a positive result at the baseline screen (*p* < 0.0001). The frequency of positive gFOBT results increased with age among both males (*p* < 0.0001) and females (*p* < 0.0001).

### 3.2. Subsequent gFOBT Screen

Among those with a normal result and no diagnosis of CRC within 24 months following the baseline gFOBT, 101,526/294,329 (34.49%) completed a subsequent gFOBT, within 21 to 30 months following the baseline. Females comprised 57,164/101,526 (56.36%) of those completing a subsequent screen (Table 2). In all age groups, males were more likely than females to receive a positive result at the subsequent screen (*p* < 0.0001, except at ages 70–74, *p* = 0.0004), and the frequency of positive results increased with age among males (*p* = 0.06) and females (*p* = 0.01).

### 3.3. CRC Diagnosed within 24 Months following Positive gFOBT

Within 24 months following a positive gFOBT at the baseline screen, males were more likely than females to receive a diagnosis of CRC: among males 501/7,390 (6.78%) compared to 261/5,737 (4.55%) among females, *p* < 0.0001, and the percent of positives diagnosed with CRC increased with age among males (*p* < 0.0001) and females (*p* < 0.0001). Following the subsequent screen, the percent of positives receiving a diagnosis of CRC was slightly lower and more likely among males 106/2,072 (5.12%) compared to females 65/1,771 (3.67%), *p* = 0.03, increasing with age among males (*p* < 0.0001) and among females (*p* = 0.006).

### 3.4. Interval CRC (CRC Diagnosed within 24 Months following Normal gFOBT)

Interval CRC occurred following baseline gFOBT screen among 254/130,821 (0.19%) of male screenees with a normal result and 227/163,508 (0.14%) of females (*p* = 0.0002), increasing in frequency with age among both males (*p* < 0.0001) and females (*p* < 0.0001). Following the subsequent screen, interval CRCs were observed among 89/42,290 (0.21%) of male screenees with a normal result and 88/55,393 (0.16%) of females (*p* = 0.06), increasing with age among both males (*p* = 0.03) and females (*p* = 0.02).

Within 24 months following the baseline gFOBT screen, 481/1,243 (38.70%) of CRCs occurring among those screened were interval CRCs, as were 177/348 (50.86%) following the subsequent screen (Table 3). Among females, a higher percent of CRCs diagnosed within 24 months following the baseline gFOBT were interval CRCs (227/488, 46.52%), compared to males (254/755, 33.64%), *p* < 0.0001, and also following the subsequent gFOBT screen, among females (88/153, 57.52%) and among males (89/195, 45.64%), *p* = 0.03. The anatomic site of CRC (distal versus other) varied among males and females by positive versus normal gFOBT result following the baseline screen (*p* = 0.0001) and the subsequent screen (*p* = 0.07).

### 3.5. Risk of Death due to Any Cause following Interval Cancer

 Screenees with an interval CRC have a higher risk of death due to any cause, compared to screenees with CRC diagnosed within 2 years following a positive result, adjusting for age, sex, and anatomic site of CRC, following the baseline (hazard ratio (HR) = 1.65, 95% confidence interval (CI) 1.32, 2.05) as well as the subsequent gFOBT screen (HR = 1.71, 95% CI 1.00, 2.91) (Table 4). In each analysis, distal anatomic site of CRC within the colon was associated with a decreased risk of death. Although more CRCs diagnosed among females were interval CRCs, the risk of death due to any cause among females did not differ from males. Among the combined cohort of cancers diagnosed after either the baseline or subsequent screen, the interaction between baseline or subsequent screen and normal or positive result was not significant (*p* = 0.68).

## 4. Discussion

Overall, 38.70% of persons diagnosed with CRC following the baseline screen and 50.86% following the subsequent screen were diagnosed with interval CRC. The increased proportion of interval cancers among CRCs diagnosed following subsequent gFOBT screening is consistent with findings from the Nottingham randomized trial [2] and the Scottish gFOBT demonstration pilot [3].

The percent of persons with a positive result who received a diagnosis of colorectal cancer within 24 months decreased from 5.8% after the baseline gFOBT to 4.45% after the subsequent gFOBT; however, the percent of those with a normal result who developed CRC within 24 months did not decrease from the baseline (0.16%) to the subsequent screen (0.18%). This was also observed by Steele et al. [3], who interpret this as showing that sequential screening reduces the prevalence of CRC susceptible to detection by gFOBT (i.e., following positive gFOBT) but cannot reduce the prevalence of CRC not susceptible (i.e., following normal gFOBT).

A higher proportion of CRCs diagnosed among female participants in gFOBT screening (46.52% at baseline and 57.52% at subsequent screen) are interval cancers, compared to the proportion among male participants (33.64% at baseline and 45.64% at subsequent screen). This is consistent with the observations of Gill et al. [4] and of Steele et al. [3]. It is not clear why females would have a higher proportion of interval cancers. Although female screenees overall are less likely to receive a positive gFOBT result compared to males, it is uncertain whether this reflects a biologic difference between females and males in the propensity of colorectal cancers to bleed.

The elevated risk of death following diagnosis of interval CRC may relate to biological differences among CRCs, or to delayed diagnosis and treatment at a more advanced stage, or a combination of both. However, Gill et al. [8] have observed that Dukes' Stage C and D CRCs have a better prognosis if diagnosed following a positive gFOBT screen compared to interval CRC with those stages.

Among the combined cohort of screenees with CRC diagnosed within 24 months of the baseline plus the subsequent gFOBT screen, we did not find a significant interaction between baseline or subsequent screen and the result of the screen. This suggests that persons with CRC diagnosed after a positive result following the subsequent screen do have a better prognosis than interval CRC following the subsequent screen, despite having had a negative screen result at the preceding screen.

We could not examine interval CRC by stage because data on stage for CRC in the OCR database are incomplete. Participation in the subsequent gFOBT screen was low and therefore the power to examine factors associated with interval CRC at subsequent screening was reduced. We could not calculate the rate of interval CRC using the proportional incidence rate recommended by Moss et al. [2] because of the background of opportunistic colorectal screening by colonoscopy in the underlying population. Nevertheless, the proportion of CRC diagnosed among screenees as interval cancers is similar to those reported by others [3].

Fecal immunochemical testing (FIT) has been adopted recently by some jurisdictions and health care agencies, on the basis of superior test characteristics. Although the published literature on interval CRC following FIT is small, the rate appears to be lower than that with gFOBT screening in two studies. Kapidzic et al. [9] reported 2/4143 (0.05%) participants with a normal result at the baseline FIT screen receiving a diagnosis of interval CRC and 2/3634 (0.06%) at the second FIT screen. Chiang et al. [10] found interval CRC in 286/349,726 (0.08%) among males and in 302/574,052 (0.05%) among females, at an initial round of FIT screening.

## 5. Conclusion

Persons participating in a colorectal screening program who receive a diagnosis of an interval CRC have a higher risk of death due to any cause compared to those with CRC diagnosed following a positive gFOBT result.

## Figures and Tables

**Table 1:** Baseline gFOBT screen between April 2008 and December 2009.

	Males	Females
Total baseline FOBT screen participants	138,211/307,456 (44.95%)	169,245/307,456 (55.05%)
Normal result	130,821 (94.65%)	163,508 (96.61%)
Positive result	7,390 (5.35%)	5,737 (3.39%)

Results by age strata^*∗*^		
50–54 years		
Normal result	38,244/40,300 (94.90%)	50,261/51,885 (96.87%)
Positive result	2,056/40,300 (5.10%)	1,624/51,885 (3.13%)
55–59 years		
Normal result	29,869/31,487 (94.86%)	37,463/38,670 (96.88%)
Positive result	1,618/31,487 (5.14%)	1,207/38,670 (3.12%)
60–64 years		
Normal result	26,610/28,109 (94.67%)	32,339/33,480 (96.59%)
Positive result	1,499/28,109 (5.33%)	1,141/33,480 (3.41%)
65–69 years		
Normal result	20,479/21,687 (94.43%)	24,775/25,703 (96.39%)
Positive result	1,208/21,687 (5.57%)	928/25,703 (3.61%)
70–74 years		
Normal result	15,619/16,628 (93.93%)	18,670/19,507 (95.71%)
Positive result	1,009/16,628 (6.07%)	837/19,507 (4.29%)

^*∗*^Difference in percent positive results between males and females in all age strata, *p* < 0.0001.

**Table 2:** Subsequent gFOBT screen 21–30 months after baseline normal gFOBT result.

	Males	Females
Total subsequent gFOBT screen participants	44,362/101,526 (43.70%)	57,164/101,526 (56.30%)
Normal result	42,290/44,362 (95.33%)	55,393/57,164 (96.90%)
Positive result	2,072/44,362 (4.67%)	1,771/57,164 (3.10%)

Results by age strata^*∗*^		
50–54 years		
Normal result	11,347/11,924 (95.16%)	16,391/16,867 (97.18%)
Positive result	577/11,924 (4.84%)	476/16,867 (2.82%)
55–59 years		
Normal result	9,705/10,140 (95.71%)	12,689/13,072 (97.07%)
Positive result	435/10,140 (4.29%)	383/13,072 (2.93%)
60–64 years		
Normal result	9,497/9,937 (95.57%)	12,057/12,461 (96.76%)
Positive result	440/9,937 (4.43%)	404/12,461 (3.24%)
65–69 years		
Normal result	8,024/8,443 (95.04%)	9,804/10,147 (96.62%)
Positive result	419/8,443 (4.96%)	343/10,147 (3.38%)
70–74 years		
Normal result	3,717/3,918 (94.87%)	4,452/4,617 (96.43%)
Positive result	201/3,918 (5.13%)	165/4,617 (3.57%)

^*∗*^Difference in percent positive results between males and females in all age strata, *p* < 0.0001, except ages 70–74, *p* = 0.0004.

**Table 3:** CRC diagnosed within 24 months following gFOBT by baseline and subsequent screen, gFOBT result, sex, and anatomic CRC site.

	CRC following positive gFOBT	CRC following normal gFOBT (interval cancer)
Baseline gFOBT screen		
CRC following baseline screen	762/1,243 (61.30%)^*∗*^	481/1,243 (38.70%)^*∗*^
CRC among males following baseline screen	501/755 (66.36%)^*∗*^	254/755 (33.64%)^*∗*^
CRC site among males		
Distal anatomic CRC site	331/501 (66.07%)^∧^	164/254 (64.57%)^∧^
Another anatomic CRC site	170/501 (33.93%)^∧^	90/254 (35.43%)^∧^
CRC among females following baseline screen	261/488 (53.48%)^*∗*^	227/488 (46.52%)^*∗*^
CRC site among females		
Distal anatomic CRC site	137/261 (52.49%)^∧^	107/227 (47.14%)^∧^
Another anatomic CRC site	124/261 (47.51%)^∧^	120/227 (52.86%)^∧^

Subsequent gFOBT screen		
CRC following subsequent screen	171/348 (49.14%)^*∗*^	177/348 (50.86%)^*∗*^
CRC among males following subsequent screen	106/195 (54.36%)^*∗*^	89/195 (45.64%)^*∗*^
CRC site among males		
Distal anatomic CRC site	75/106 (70.75%)^∧^	57/89 (64.04%)^∧^
Another anatomic CRC site	31/106 (29.25%)^∧^	32/89 (35.96%)^∧^
CRC among females following subsequent screen	65/153 (42.48%)^*∗*^	88/153 (57.52%)^*∗*^
CRC site among females		
Distal anatomic CRC site	38/65 (58.46%)^∧^	51/88 (57.95%)^∧^
Another anatomic CRC site	27/65 (41.54%)^∧^	37/88 (42.05%)^∧^

^*∗*^Row percent.

^∧^Cell percent.

**Table 4:** Death from any cause following CRC by baseline and subsequent gFOBT screens.

Variable	CRC ≤ 24 months following baseline gFOBT	CRC ≤ 24 months following subsequent gFOBT
	Hazard ratio (95% confidence interval)	Hazard ratio (95% confidence interval)
Positive gFOBT result	Reference	Reference
Normal gFOBT result	1.65 (1.32, 2.05)	1.71 (1.00, 2.91)

Female	0.85 (0.68, 1.07)	0.79 (0.46, 1.34)
Male	Reference	Reference

Distal anatomic CRC site	0.75 (0.61, 0.94)	0.41 (0.24, 0.70)
Another anatomic CRC site	Reference	Reference

Age, per one-year increase	1.04 (1.02, 1.05)	1.02 (0.97, 1.06)
